# Gut microbiota mediated the therapeutic efficiency of Simiao decoction in the treatment of gout arthritis mice

**DOI:** 10.1186/s12906-023-04042-4

**Published:** 2023-06-21

**Authors:** Xiaoying Lin, Mingzhu Wang, Zhixing He, Guifeng Hao

**Affiliations:** 1 Department of Plastic Surgery, Sir Run Run Shaw Hospital, Zhejiang University School of Medicine, Hangzhou, 310016 China; 2 Institute of Basic Research in Clinical Medicine, School of Basic Medical Science, Zhejiang Chinese Medical University, Hangzhou, 310053 China; 3 Center for General Practice Medicine, Department of Rheumatology and Immunology, Zhejiang Provincial People’s Hospital (Affiliated People’s Hospital, Hangzhou Medical College), Hangzhou, 310014 China

**Keywords:** Gout arthritis, Simiao decoction, Gut microbiota, Fecal microbiota transplantation, Allopurinol

## Abstract

**Background:**

Gut microbiota plays a significant role in the development and treatment of gouty arthritis. Simiao decoction has been shown to alleviate gouty arthritis by inhibiting inflammation, regulating NLRP3 inflammasome, and altering gut microbiota. However, there is no evidence to prove whether gut microbiota directly mediates the therapeutic efficiency of Simiao decoction in treating gout arthritis.

**Methods:**

In this study, fecal microbiota transplantation (FMT) was used to transfer the gut microbiota of gout arthritis mice treated with Simiao decoction or allopurinol to blank gout arthritis mice, in order to investigate whether FMT had therapeutic effects on gout arthritis.

**Results:**

Both Simiao decoction and allopurinol effectively reduced the levels of serum uric acid, liver XOD activity, foot thickness, serum IL-1β, and G-CSF in gout arthritis mice. However, Simiao decoction also had additional benefits, including raising the pain threshold, reducing serum TNF-α and IL-6, alleviating gut inflammation, and repairing intestinal pathology, which were not observed with allopurinol treatment. Moreover, Simiao decoction had a greater impact on gut microbiota than allopurinol, as it was able to restore the abundance of phylum Proteobacteria and genus *Helicobacter*. After transplantation into gout arthritis mice, gut microbiota altered by Simiao decoction exhibited similar therapeutic effects to those of Simiao decoction, but gut microbiota altered by allopurinol showed no therapeutic effect.

**Conclusions:**

These findings demonstrates that Simiao decoction can alleviate gout arthritis symptoms by regulating gut microbiota.

**Supplementary Information:**

The online version contains supplementary material available at 10.1186/s12906-023-04042-4.

## Introduction

Gout is a prevalent type of inflammatory arthritis that occurs due to the deposition of monosodium urate (MSU). This condition is directly linked to hyperuricemia, which is caused by the disruptions in purine metabolism and/or decreased uric acid excretion [1]. In Mainland China, the prevalence of gout is 1.6%, with higher rates observed in males (1.9%) than in females (0.5%) [2]. Gout arthritis is characterized by recurrent attacks and typically involve joint inflammation, including redness, swelling, heat, and pain, with acute attacks causing severe pain [3]. The inflammatory cascade triggered by MSU crystals is the main cause of the acute symptoms of gout, and the NLRP3 inflammasome pathway is primarily responsible for MSU-induced cellular inflammation [4]. MSU crystal deposition in the joints activates the NLRP3 inflammasome, leading to the maturation and release of cytokines such as IL-1β and IL-18, results in the gout arthritis flare-ups [4].

Cumulative research has consistently demonstrated the disturbances in gut microbiota among gout patients [5, 6] and animal models [7, 8]. Furthermore, modifying gut microbiota has been found to affect gout arthritis symptoms [9, 10]. The reason for this may be attributed to gut microbiota’s role in regulating the activation of NLRP3 inflammasome [11, 12] and uric acid metabolism [13, 14]. Severe drugs used in gout arthritis therapy have been shown to effectively alter the gut microbiota [15, 16]. Simiao decoction, a classical traditional Chinese medicine formula, is widely used for gout arthritis therapy in clinical practice [17]. It has been reported to be both effective and safe in treating gout patients [18–20]. In gout arthritis or hyperuricemia mouse models, Simiao decoction can reduce renal urate transporters, enhance antioxidant enzymes activities, inhibit NLRP3 inflammasome, and decrease serum UA and liver XOD levels [21–23]. In our previous study, we demonstrated that Simiao decoction can alleviate gout arthritis symptoms by modulating pro-inflammatory cytokines and gut microbiota in gouty arthritis mice [24]. However, the previous study only established a correlation between gut microbiota and the therapeutic effect of Simiao decoction in treating gout arthritis, it did not prove evidence of the direct involvement of gut microbiota in Simiao decoction’s efficacy against gout arthritis.

To further clarify the role of gut microbiota in treating gout arthritis by Simiao decoction, we conducted a fecal microbiota transplantation (FMT) study by transplanting fresh feces from Simiao decoction-treated gout mice into untreated gout arthritis mice. This allowed us to examine the therapeutic effects of Simiao decoction-altered gut microbiota. Allopurinol was selected as the control drug for Simiao decoction, since it was a well-established and widely used medication for gout arthritis treatment in clinical trials.

## Materials and methods

### Animals

Male specific pathogen-free (SPF) C57BL/6 mice (4 weeks old, weighing 15 ± 3 g) were purchased from Shanghai SLAC Laboratory Animal Co., Ltd. and housed in the Zhejiang Chinese Medical University laboratory animal research center under SPF conditions. All animal experiments were approved by the Ethics Committee of Zhejiang Chinese Medical University (approval number 20210816-12). The mice were maintained under a 12 h/12 h light/dark cycle with constant temperature (25 ± 1 °C) and humidity (50 ± 5%), with ad libitum access to food and water. All protocols in this study were carried out in accordance with relevant guidelines and regulations for using animals in compliance with the ARRIVE guidelines (https://arriveguidelines.org).

### Preparation of Simiao decoction

Simiao decoction was composed of *Atractylodes lancea* (Thunb.) DC. (12 g), *Phellodendron amurense Rupr*. (12 g), *Achyranthes bidentata* Blume (12 g) and *Coix lacryma-jobi* var. ma-yuen (Rom.Caill.) Stapf (30 g), which were purchased from Zhejiang Chinese Medical University Medicine Yinpian Factory (Hangzhou, China) and authenticated by the authors. A voucher specimen (No.20,200,305) has been deposited at the Institute of Basic Research in Clinical Medicine of Zhejiang Chinese Medical University (Hangzhou, China).

### Study design

**Fig. 1:**
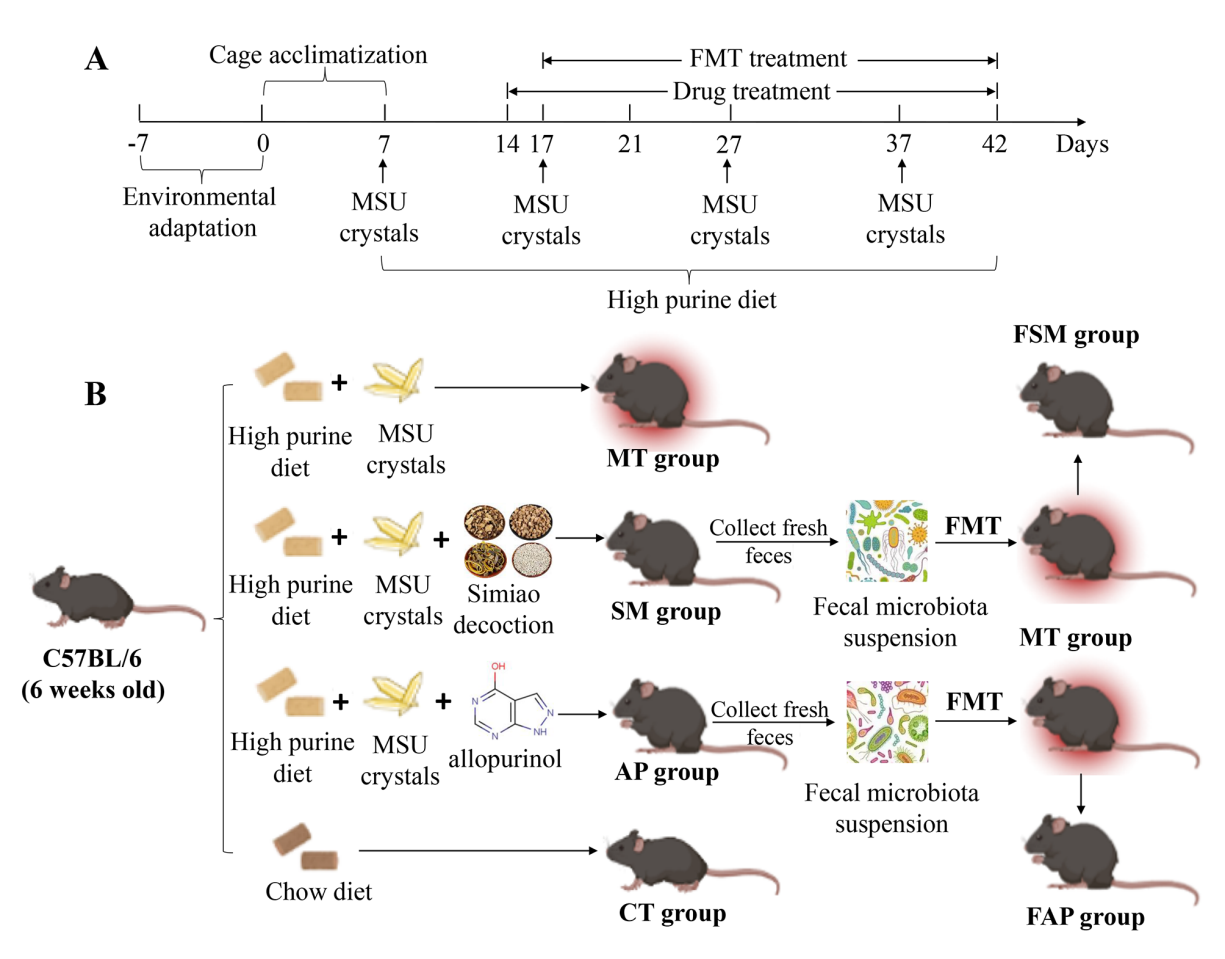
Overview of experimental schedule (**A**) and grouping information (**B**) in the study. CT: control mice; MT: gout arthritis mice; SM: gout mice treated with Simiao decoction; AP: gout mice treated with allopurinol; FSM: gout mice transplanted with fecal microbiota from Simiao decoction-treated gout mice; FAP: gout mice transplanted with fecal microbiota from allopurinol-treated gout mice; FMT, fecal microbiota transplantation; MSU, monosodium urate crystal

After 7 days of environmental adaptation and 7 days of cage acclimatization, male C57BL/6 mice (6 weeks old) were randomly assigned to six groups (N = 6 mice/group): (1) control group (CT) fed a normal diet; (2) gout arthritis group (MT) was fed a high purine diet (10% yeast extract) and received injections of monosodium urate (MSU) crystals (25 mg/mL in PBS solution/mouse per 10 days) into the right foot joint for a duration of 35 days; (3) Simiao decoction group (SM), where gout arthritis mice were orally administered 8.0 g/kg of Simiao decoction daily after the establishment of the gout arthritis model from day 14; (4) FSM group, where gout arthritis mice were given 200 µL/day aliquot of fresh fecal suspensions daily (collected from the mice of SM group daily) from day 17; (5) allopurinol group (AP), where gout arthritis mice were orally administered 30 mg/kg of allopurinol daily after the establishment of gout arthritis model from day 14; and (6) FAP group, where gout arthritis mice were given 200 µL/day aliquot of fresh fecal suspensions daily (collected from the mice of AP group daily) from day 17. The experimental flow and grouping information were presented in Fig. 1.

Simiao decoction was prepared according to the method described by Lin et al. [24]. The dosage of Simiao decoction for adult is 66 g (total raw materials)/day, and for mice, it is 8.0 g/kg/day, calculated by converting the human dosage to that of mice according to their respective body surface areas, in accordance with Chinese Medicine Pharmacology Research Technology. Fresh feces were collected daily from donor mice as follows: The mouse was fixed and its tail was lift. The lower abdomen of the mouse was pressed with fingers, and the fresh feces were collected in a sterile micro-centrifuge tube. The fecal suspensions were resuspended in 5 times (weight/volume) phosphate-buffered saline solution, passed through a 20-mm filter to remove large particulate, and transferred into the receptor mice. Throughout the trial, all mice were weighed each week, and the drug doses were adjusted accordingly.

At the end of the trial (day 42), the mice were anaesthetised with 3% pentobarbital sodium at a dosage of 40 mg/kg via intraperitoneal injection and sacrificed using cervical dislocation. Blood samples were obtained from the eye socket vein of each mouse, then centrifuged at 3000 rpm for 10 min at 4 ^o^C for serum. Fecal material was removed from the colon and stored at -80 °C for further analysis. Colon tissues were separated into two sections, one part was fixed in 4% formalin for histological analysis, while the other was rapidly snap-frozen in liquid nitrogen and stored at -80 °C for the determination of proinflammatory cytokines. Moreover, liver tissues were also immediately snap-frozen in liquid nitrogen and stored at -80 °C until assessing xanthine oxidase (XOD) activity.

### MSU crystal preparation and injection

The method for preparing of MSU crystals followed previously published protocols [7]. Monosodium urate (800 mg) was dissolved in 155 mL of boiling milli-Q water containing 5 mL of NaOH. The solution was adjusted to pH 7.2 and cooled gradually by constant stirring at room temperature. The crystals were collected by centrifugation at 3000 g for 2 min at 4 °C), then evaporated and sterilized by heating at 180 °C for 2 h. The MSU crystals were stored in sterile microtubes until use.

To induce joint inflammation, each mouse was injected with MSU (1.0 mg/40 µL) into the right foot joint via intra-articular (i.a.) injection under isoflurane anesthesia. As a blank control, 40 µL of PBS solution was injected into the right foot joint.

### Evaluation of foot thickness and pain threshold

The thickness of each mouse’s foot was measured using a caliper (Meinaite, Germany) before the stimulus and 48 h after i.a. administration of MSU, with foot swelling was expressed as the ratio of the difference between the thickness at 48 h and the thickness at time zero. To quantify foot pain, the mechanical withdrawal threshold (MWT) was measured using von Frey hairs (Stoelting, Wood Dale, IL) and the up-and-down method [25]. Mice were individually placed in testing chambers with metal mesh floors for at least 30 min for acclimation before testing in a quiet, temperature-controlled room. Each test began with a force of 0.16 g delivered perpendicularly to the central plantar surface of either hind paw for 3 s with a hair. Positive responses included sudden paw withdrawal, flinching, or paw licking, and no response was considered negative. The threshold force required to elicit withdrawal (50% hind paw withdrawals) was determined for right hind paws [26]. The minimum force that could provoke at least three withdrawal responses of the right hind paw was defined as the MWT. All behavioral tests were performed by an investigator who was blinded to the experimental design.

### Assessment of serum, colon, and liver indexes

Serum uric acid and liver XOD concentrations were measured using assay kits (Nanjing Jiancheng Bioengineering Institute, Nanjing, China), following the manufacturer’s instructions. Colon or liver tissues were homogenized in buffer solution (0.05 g/1 mL) using a high-speed homogenizer and centrifuged at 12,000 rpm at 4 °C for 10 min. The concentrations of IL-1β, IL-6, G-CSF, and TNF-α in serum and colon supernatants were quantified using assay kits (CUSABIO, Wuhan, China), following the manufacturer’s protocols.

### Gut microbiota analysis

Total genomic DNA was isolated from each stool sample using a stool DNA isolation kit (Qiagen, Hilden, Germany) and quantified using a NanoDrop 2000 spectrophotometer (Thermo Fisher Scientific, USA). The qualified DNA was sequenced for the V3-V4 region of the 16 S rRNA gene using an Illumina Miseq platform (Illumina, San Diego, CA, USA) at Shanghai Personalbio Technology Co., Ltd. The raw data were deposited in the National Center for Biotechnology Information (NCBI) Sequence Read Archive (SRA) database (ID: PRJNA932929). Microbiome data were analyzed using the Genescloud platform (http://greengenes.secondgenome.com/). The Quantitative Insights Into Microbial Ecology (QIIME) software was used for data assembly, demultiplexing, and quality filtering. Sequences with ≥ 97% similarity were assigned to the same operational taxonomic unit (OTU), and taxonomic assignment was done using the Greengenes database. Principal Coordinates Analysis (PCoA) was conducted to compare microbiota compositions between different samples. Linear discriminant analysis effect size (LEfSe) and one-way ANOVA analysis were performed to identify statistically significant genera between the two groups.

#### Western blot analysis

Colon samples were homogenized in RIPA lysis buffer (Beyotime Biotechnology, Shanghai, China), supplemented with a cocktail of protease and phosphatase inhibitor, and incubated on ice for 30 min. The homogenates were then centrifuged at 12,000×g for 10 min to obtain total proteins, and their concentrations were determined using the BCA Protein Assay Kit (Beyotime biotechnology, Shanghai, China). Subsequently, 40 µg of cell lysates were separated by SDS-PAGE on a 7.5/10% gel and transferred onto a nitrocellulose membrane (Pall Corporation, MI, USA). The membranes were blocked with 5% BSA in TBST (Tris-buffered saline with 0.1% Tween-20) at room temperature for 1 h to minimize non-specific binding sites, followed by overnight incubation with primary antibodies at 4 °C, and then incubated with the appropriate secondary antibody. The Western blot results were scanned and analyzed using an infrared imaging system (Odyssey CLx; LI-COR Biosciences). Gel quantification was performed using ImageJ software.

Commercially available antibodies were used as follows: NLRP3 (1:1000 dilution; Cell Signaling Technology, #15,101), ASC (1:1000 dilution; Cell Signaling Technology, #67,824), Caspase-1 (1:1000 dilution; Abcam, #ab179515), β-Actin (1:5000; R&D system, #MAB8929), Dylight 800, Goat anti-Rabbit IgG (1:10000 dilution; Immunoway, #RS23920), and Dylight 680, Goat anti-Mouse IgG (1:10000 dilution; Immunoway, #RS23710).

### Histological analysis

Formalin-fixed colon sections of each mouse were sectioned for histopathological analysis. H&E and AB-PAS staining were used to stain the tissue sections according to the manufacturer’s instructions (Beyotime Biotechnology, Shanghai, China). The images were evaluated using ImageJ software, and the intestinal villus height, intestinal wall thickness, and goblet cell quantity were counted and compared among groups.

### Statistical analysis

Statistical analyses were performed using SPSS software version 22.0. One-way ANOVA or the rank-sum test was used for data analysis based on the normality test and homogeneity of variance. P-values were adjusted using the Benjamini-Hochberg method to control the false discovery rate (FDR), and an adjusted *p*-value < 0.05 was considered statistically significant.

## Results

### Effects of Simiao decoction, allopurinol, or FMT on gut microbiota

**Fig. 2:**
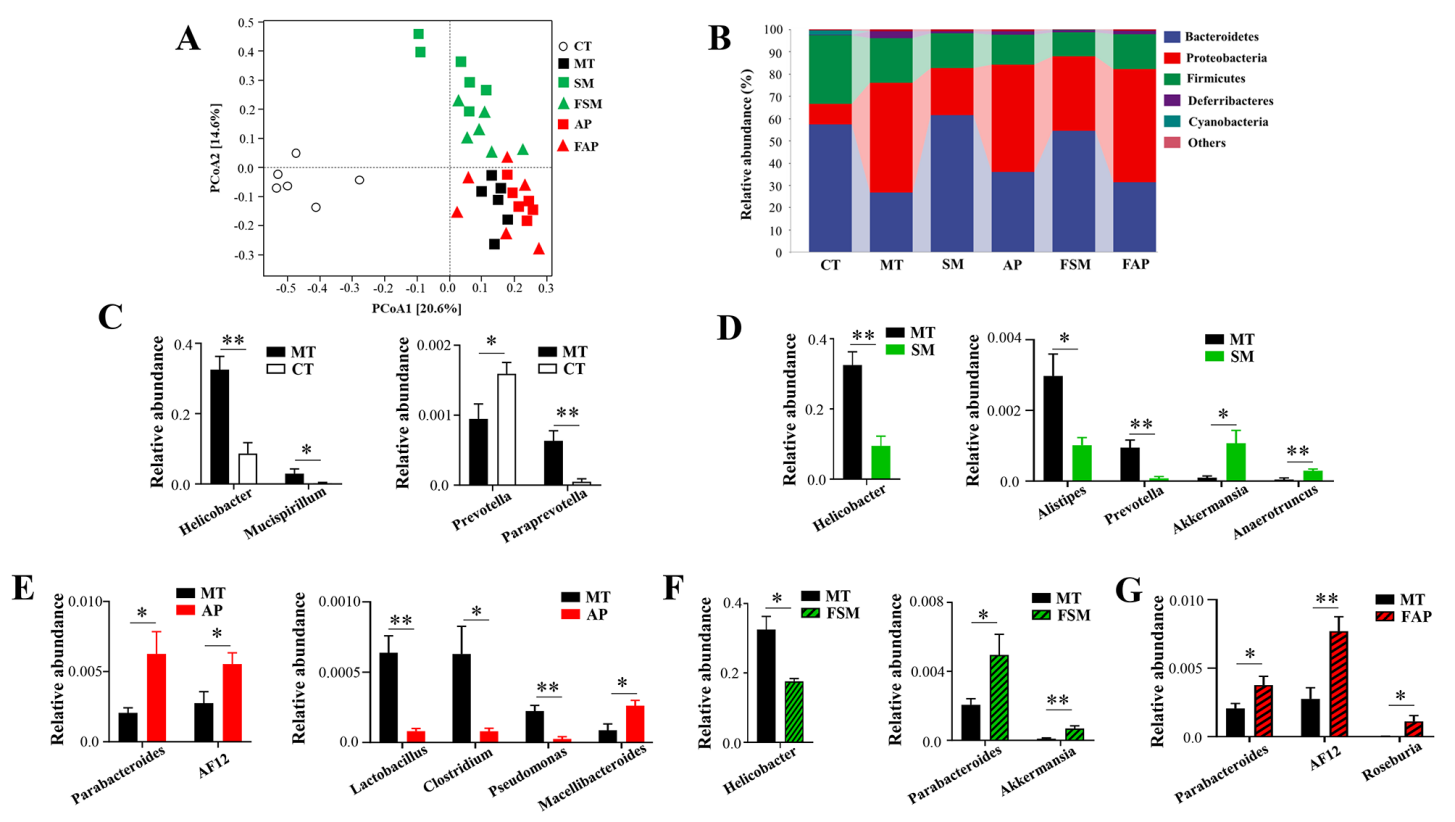
Alterations in gut microbiota induced by Simiao decoction, allopurinol, or FMT. (**A**) PCoA score plot of gut microbiota based on UniFrac distance matrices; (**B**) The percentage abundance of major phylum; Significantly different genera between CT and MT (**C**), between MT and SM (**D**), between MT and AP (**E**), between MT and FSM (**F**), and between MT and FAP (**G**). CT: control mice; MT: gout arthritis mice; SM: gout mice treated with Simiao decoction; AP: gout mice treated with allopurinol; FSM: gout mice transplanted with fecal microbiota from Simiao decoction-treated gout mice; FAP: gout mice transplanted with fecal microbiota from allopurinol-treated gout mice. “**” indicates *p* < 0.01; “*” indicates *p* < 0.05. N = 6 mice/group

A scatter plot based on PCoA scores (Fig. 2A) demonstrated distinct microbial compositions in the comparisons of CT vs. MT, MT vs. SM, and MT vs. FSM, with some overlap observed among MT, AP, and FAP groups. At the phylum level, gout arthritis mice had higher levels of Proteobacteria and lower levels of Bacteroidetes and Firmicutes compared to control mice (Fig. 2B). Simiao decoction and its related FMT successfully restored the dysbiosis of microbial phyla in gout arthritis mice, whereas allopurinol and its related FMT did not show any effect (Fig. 2B). Therefore, Simiao decoction had a more significant impact on gut microbiota of gout arthritis mice compared to allopurinol.

Furthermore, a combination of LEfSe and one-way ANOVA analyses revealed significant differences in microbial genera between two groups. As shown in Fig. 2C, MT group had higher levels of genus *Helicobacter*, *Mucispirillum*, and *Paraprevotella* and a lower level of genus *Prevotella* than the CT group. Treatment of Simiao decoction reduced the abundances of genus *Helicobacter*, *Alistipes*, and *Prevotella* and increased the abundances of genus *Akkermansia* and *Anaerotruncus* in gout arthritis mice (Fig. 2D). The genera altered by Simiao decoction differed from those altered by allopurinol. Allopurinol increased the abundances of genus *Parabacteroides*, AF12, and *Macellibacteroides* and reduced the abundances of genus *Lactobacillus*, *Clostridium*, and *Pseudomonas* in the treatment of gout arthritis mice (Fig. 2E). In addition, FMT caused alterations in microbial genera in gout arthritis mice. The transplantation of gut microbiota derived from Simiao decoction-treated mice reduced genus *Helicobacter* and increased genus *Parabacteroides* and *Akkermansia* in gout arthritis mice (Fig. 2F), while the transplantation of gut microbiota derived from allopurinol-treated mice increased genus AF12, *Parabacteroides*, and *Roseburia* in gout arthritis mice (Fig. 2G).

### Effects of Simiao decoction, allopurinol, or FMT on gout arthritis symptoms

**Fig. 3:**
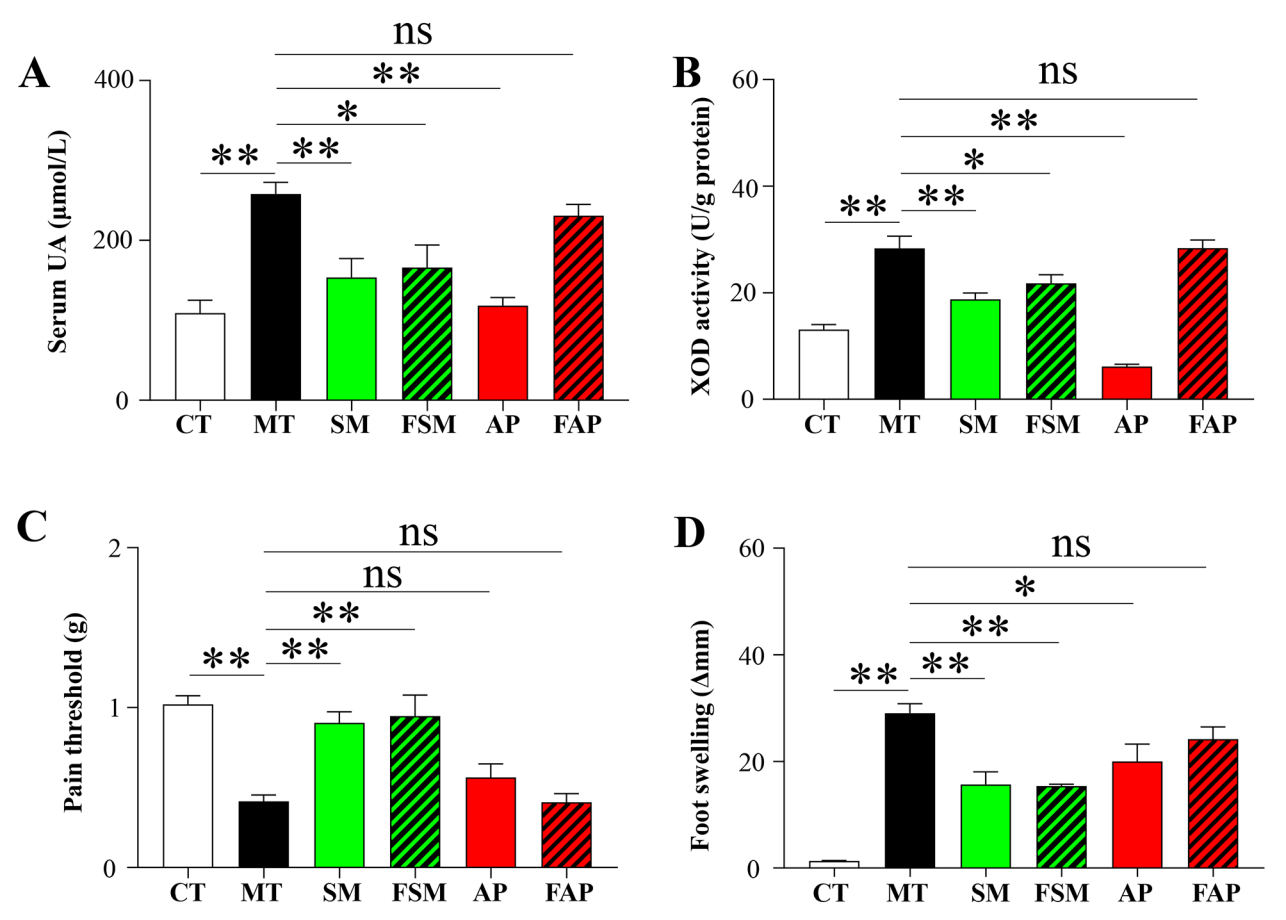
Differences in serum levels of uric acid (**A**), liver xanthine oxidase activity (**B**), pain threshold (**C**), and foot swelling (**D**) among different groups. CT: control mice; MT: gout arthritis mice; SM: gout mice treated with Simiao decoction; AP: gout mice treated with allopurinol; FSM: gout mice transplanted with fecal microbiota from Simiao decoction-treated gout mice; FAP: gout mice transplanted with fecal microbiota from allopurinol-treated gout mice. “**” indicates *p* < 0.01; “*” indicates *p* < 0.05. N = 6 mice/group

Feeding a high purine diet could significantly increase serum uric acid and liver XOD activity, and the injection of MSU crystals induced foot swelling and reduced pain threshold in mice with gouty arthritis (Fig. 3). Simiao decoction and its related FMT significantly alleviated all four gout arthritis symptoms (Fig. 3). Allopurinol was also effective in reducing serum uric acid, liver XOD activity, and foot swelling in gout arthritis mice (Fig. 3A C). However, the transplantation of gut microbiota derived from allopurinol-treated mice showed no therapeutic efficiency in improving the four gout arthritis symptoms mentioned above (Fig. 3).

### Effects of Simiao decoction, allopurinol, or FMT on serum proinflammatory cytokines

**Fig. 4:**
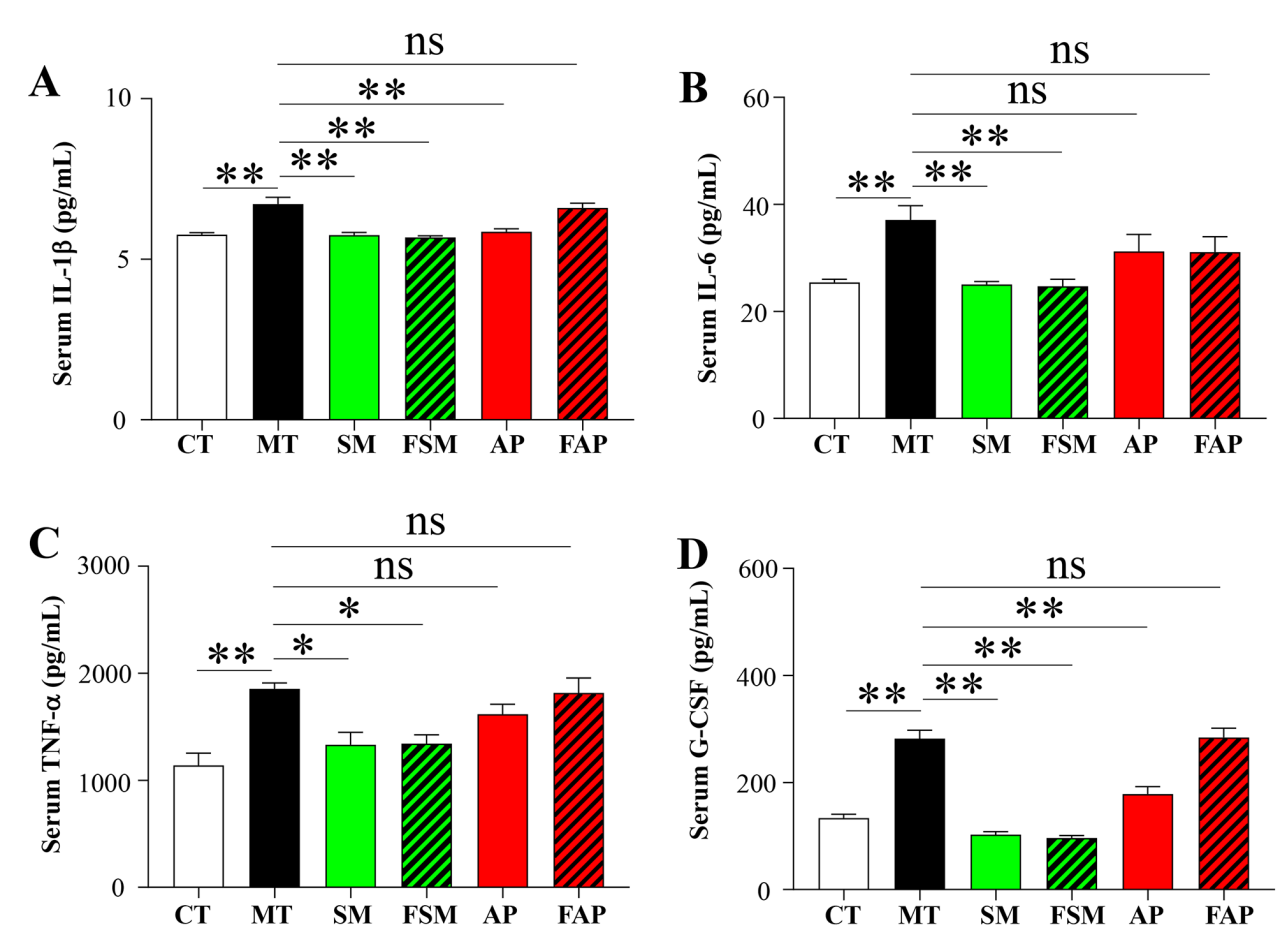
Differences in the levels of serum IL-1β (**A**), IL-6 (**B**), TNF-α (**C**), and G-CSF (**D**) among different groups. CT: control mice; MT: gout arthritis mice; SM: gout mice treated with Simiao decoction; AP: gout mice treated with allopurinol; FSM: gout mice transplanted with fecal microbiota from Simiao decoction-treated gout mice; FAP: gout mice transplanted with fecal microbiota from allopurinol-treated gout mice. “**” indicates *p* < 0.01; “*” indicates *p* < 0.05. N = 6 mice/group

This study used serum IL-1β, IL-6, TNF-α, and G-CSF levels to assess the severity of gout arthritis in mice. As depicted in Fig. 4, gout arthritis mice had a significant upregulation of the above four proinflammatory cytokines. Simiao decoction and its related FMT significantly reduced serum levels of these cytokines (Fig. 4). Allopurinol was able to significantly decrease serum levels of IL-1β, IL-6, and G-CSF, but showed no significant effect on serum TNF-α in gout arthritis mice (Fig. 4). The transplantation of gut microbiota derived from allopurinol-treated mice did not have any noticeable effect on the aforementioned serum proinflammatory cytokines in gout arthritis mice (Fig. 4).

### Effects of Simiao decoction, allopurinol, or FMT on gut inflammation

**Fig. 5:**
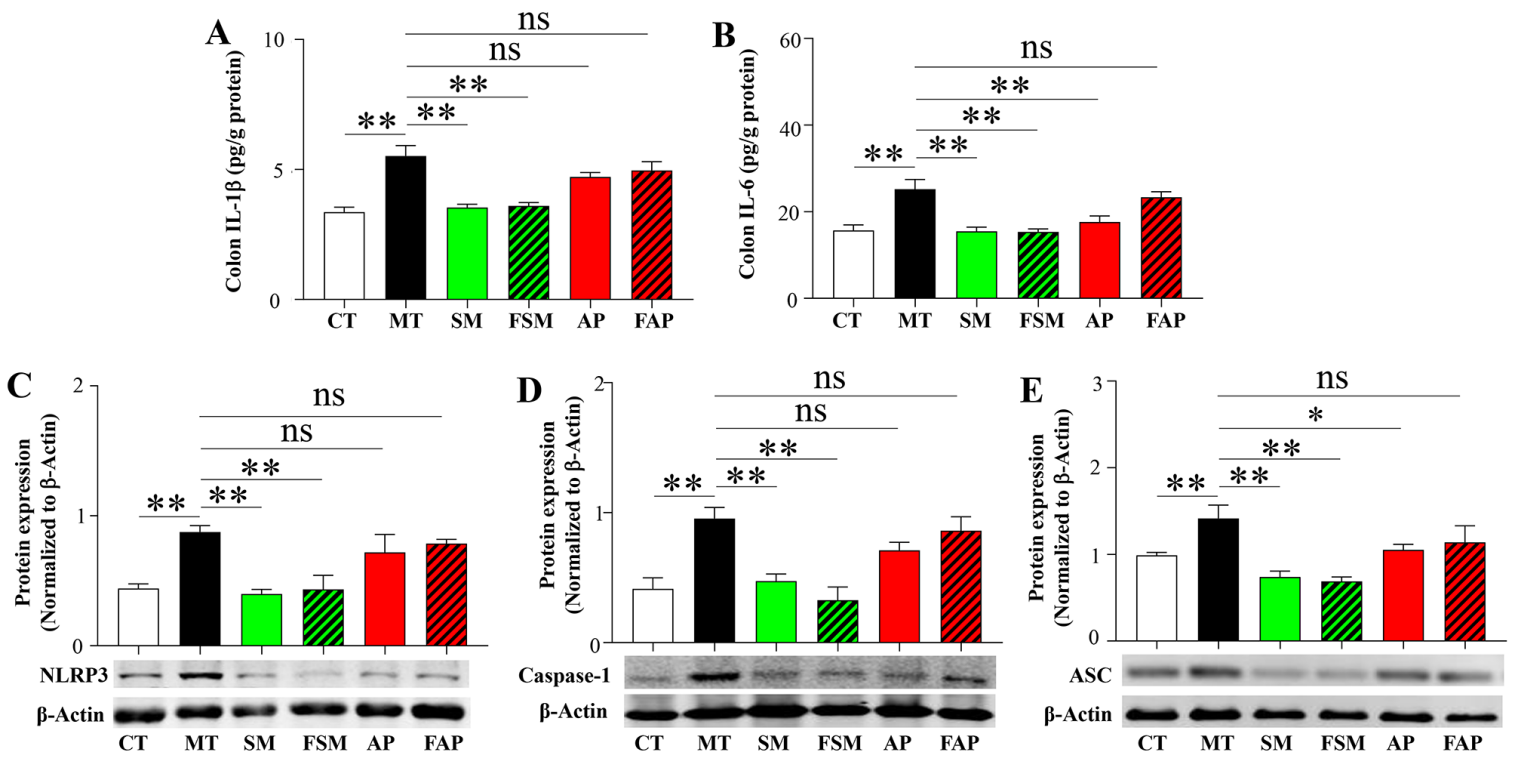
Differences in the levels of IL-1β (**A**) and IL-6 (**B**) in **t**he colon, as well as the expression of NLRP3 (**C**), Caspase-1 (**D**) and ASC (**E**) in the colon, were observed among the different groups. CT: control mice; MT: gout arthritis mice; SM: gout mice treated with Simiao decoction; AP: gout mice treated with allopurinol; FSM: gout mice transplanted with fecal microbiota from Simiao decoction-treated gout mice; FAP: gout mice transplanted with fecal microbiota from allopurinol-treated gout mice. “**” indicates *p* < 0.01; “*” indicates *p* < 0.05. N = 3 mice/group

Gut inflammation is a significant contributor to the development of gout arthritis model. As depicted in Fig. 4A and B, gout arthritis mice exhibited significantly higher concentrations of colon IL-1β and IL-6 compared to control mice. Simiao decoction and its related FMT were effective in significantly reducing the concentrations of colon IL-1β and IL-6 (Fig. 5A and B). Allopurinol showed a decreasing effect on colon IL-1β but had no significant effect on colon IL-6. However, the transplantation of gut microbiota derived from allopurinol-treated mice did not reduce the concentrations of colon IL-1β and IL-6 in gout arthritis mice (Fig. 5A and B).

To investigate the expression of NLRP3 inflammasome, colon samples were also collected. The NLRP3 inflammasome was activated in the colon of gout arthritis mice. Simiao decoction and its related FMT significantly reduced the production of NLRP3, caspase-1 and ASC, but allopurinol only reduced the production of ASC, and its related FMT had no significant influence on NLRP3 inflammasome in gouty arthritis mice (Fig. 5C and E, Supplementary information files).

### Effects of Simiao decoction, allopurinol, and FMT on colon pathology

**Fig. 6:**
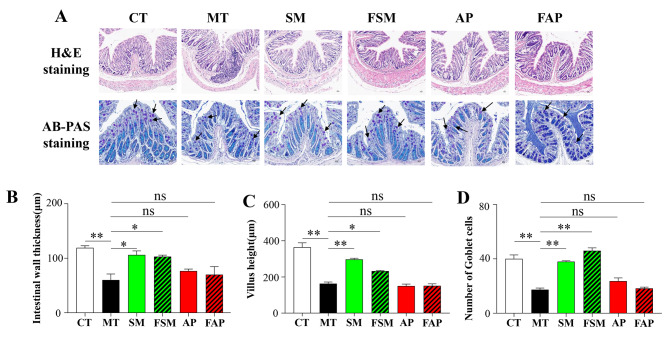
Representative images of H&E-stained and AB-PAS-stained (**A**) colon tissue histology is shown. Differences in colon intestinal wall thickness (**B**), villus height (**C**), and number of goblet cells (**D**) are observed among different groups. CT: control mice; MT: gout arthritis mice; SM: gout mice treated with Simiao decoction; AP: gout mice treated with allopurinol; FSM: gout mice transplanted with fecal microbiota from Simiao decoction-treated gout mice; FAP: gout mice transplanted with fecal microbiota from allopurinol-treated gout mice. “**” indicates *p* < 0.01; “*” indicates *p* < 0.05. N = 3 mice/group

H&E and AB-PAS staining were utilized to assess colon pathology (Fig. 6). Histological examination indicated that gout arthritis induced significant damage to the colon tissues, as evidenced by thinning of the intestinal wall, distorted crypts, loss of goblet cells, and inflammatory cell infiltration (Fig. 6). Simiao decoction and its related FMT were able to significantly restore the damage to colon tissues, as evidenced by an increase in intestinal wall thickness, villus height, and goblet cells and a reduction in inflammatory cell infiltration (Fig. 6). However, allopurinol and its related FMT did not result in any significant repair of colon tissue damage in the gout arthritis mice (Fig. 6).

## Discussion

In recent, intestinal dysbiosis associated with gout arthritis has been widely studied, since gut microbiota could mediate MSU crystal-induced inflammation [8] and hyperuricemia [13, 27]. Gut microbiota affects not only the activity of gout arthritis [28] but also the therapeutic effects of drugs on gout arthritis [29]. Previous studies have reported that Simiao decoction and allopurinol could induce alterations in gut microbiota during the treatment of gout [15, 24, 30]. However, there is still a lack of direct evidence confirming whether these treatments alleviate gout arthritis symptoms by regulating gut microbiota. This study used FMT to directly demonstrate that Simiao decoction could regulate gut microbiota to alleviate gout arthritis, while allopurinol does not have the same effect.

Allopurinol is recognized as a xanthine oxidase inhibitor that reduces uric acid levels. The present study indicates that allopurinol not only lowers uric acid levels but also exhibits an anti-inflammatory effect. This effect may be attributed to the drug’s ability to lower uric acid, which results in decreased urate crystal formation and less stimulation of NLRP3 inflammasome [31]. Compared to allopurinol, Simiao decoction not only had a similar uric acid-lowering effect but also demonstrated superior anti-inflammatory effect on both serum and gut inflammation. It is widely known that gout inflammation is a form of systemic inflammation that can occur in the joints, intestine, and kidney [32]. This study was limited in its capacity to observe inflammation levels within foot joint due to our failure to separate the tissues of foot joint.

Both Simiao decoction and allopurinol had an impact on gut microbiota in the treatment of gout arthritis mice. The PCoA analysis indicated the effect of Simiao decoction was greater than that of allopurinol in treating gout arthritis mice. The differences between the two treatments were also reflected by the alterations in microbial phylum and genus. At the phylum level, Simiao decoction was able to reverse the increase of phylum Proteobacteria in gout arthritis mice, while allopurinol had no such effect. Phylum Proteobacteria is considered a biomarker of the “pro-inflammatory” state of the host [33, 34] and has been found to be significantly elevated in gout patients compared to healthy individuals [35, 36]. At the genus level, while the number of genera altered by Simiao decoction was similar to that altered by allopurinol, Simiao decoction significantly altered the dominant genus *Helicobacter* in gout arthritis mice. Genus *Helicobacter* has been increasingly recognized as emerging pathogens over decades [37] and has been associated with the development of various diseases such as inflammatory bowel disease [38], colorectal cancer [39], and irritable bowel syndrome [40]. A significant increase in genus *Helicobacter* was observed in gout arthritis mice, while Simiao decoction was able to reduce its abundance. Genus *Helicobacter* might aggravate gout arthritis by activating NLRP3 inflammasome [41]. Furthermore, Simiao decoction reduced the abundances of genus *Alistipes* and *Prevotella* while increasing the abundance of genus *Akkermansia* and *Anaerotruncus*. Both *Alistipes* and *Prevotella* have been found to be dysregulated in gout patients [5, 42] and have been implicated in promoting chronic inflammation [43, 44]. The underlying mechanism of *Prevotella* in inducing gout inflammation might be by enhancing IL-1b production through activating NLRP3 inflammasome [45]. The increases in *Akkermansia* and *Anaerotruncus* might represent a protective mechanism associated with recovery from the disease and are not part of the pathogenic mechanism that induces or maintains the disease [46, 47]. In addition, genus *Akkermansia* could effectively attenuate hyperuricemia by regulating uric acid metabolism and inflammation [48]. Some of the genera altered by allopurinol have also been reported to be associated with gout arthritis, including *Clostridium* [49] and *Lactobacillus* [50]. However, these genera accounted for a relatively small proportion of gut microbiota in gout arthritis mice.

Subsequently, this study performed FMT to investigate whether the therapeutic effects of Simiao decoction and allopurinol on gout arthritis were mediated by gut microbiota modulation. FMT was used to determine whether the drugs exerted their therapeutic effects by modulating gut microbiota [27, 51, 52]. Results showed that FMT from Simiao decoction-treated gout mice was able to alleviate gout arthritis symptoms, whereas transplantation of gut microbiota from allopurinol-treated gout mice had no therapeutic effect. This may be due to the fact that transplantation of gut microbiota from Simiao decoction-treated gout mice was able to decrease the abundance of genus *Helicobacter* and increase the abundance of genus *Akkermansia*, which are associated with beneficial effects. On the other hand, FMT from allopurinol-treated gout arthritis mice did not significantly alter the abundance of genus *Helicobacter* or other gout-associated genera.

## Conclusion

To summarize, our data demonstrated that Simiao decoction can alleviate gout arthritis symptoms by modulating gut microbiota. This study provides direct evidence that gut microbiota can be a mechanism of traditional Chinese medicine in treating diseases. However, the findings have not been confirmed with human samples, and the underlying mechanisms from the host’s perspective have not been elucidated. Nonetheless, this research could advance our understanding of the therapeutic mechanisms of Simiao decoction.

## Electronic supplementary material

Below is the link to the electronic supplementary material.

Supplementary Material 1

## Data Availability

Raw sequencing reads of 16 S rRNA sequencing described have been deposited in the NCBI Sequence Read Archive under accession number: PRJNA932929. Further information and requests for resources and reagents should be directed to the corresponding author, Z.H. (hzx2015@zcmu.edu.cn)
