# Correction: Gut microbiota mediated the therapeutic efficiency of Simiao decoction in the treatment of gout arthritis mice

**DOI:** 10.1186/s12906-023-04090-w

**Published:** 2023-07-17

**Authors:** Xiaoying Lin, Mingzhu Wang, Zhixing He, Guifeng Hao

**Affiliations:** 1 Department of Plastic Surgery, Sir Run Run Shaw Hospital, Zhejiang University School of Medicine, Hangzhou, 310016 China; 2 Institute of Basic Research in Clinical Medicine, School of Basic Medical Science, Zhejiang Chinese Medical University, Hangzhou, 310053 China; 3 Center for General Practice Medicine, Department of Rheumatology and Immunology, Zhejiang Provincial People’s Hospital (Affiliated People’s Hospital, Hangzhou Medical College), Hangzhou, 310014 China

**Correction: BMC Complement Med Ther 23, 206 (2023)**

10.1186/s12906-023-04042-4

Following publication of the original article [1], the authors identified an error in the affiliation of author name Guifeng Hao.

The incorrect author affiliation is: Guifeng Hao^2,3^

The correct author affiliation is: Guifeng Hao^3^

The author group has been updated above and the original article has been corrected.
