# Supplementary figures and images for: Gut microbiota mediated the therapeutic efficiency of Simiao decoction in the treatment of gout arthritis mice
Source: BMC Complement Med Ther. 2023 Jun 21;23:206. doi: 10.1186/s12906-023-04042-4 (PMC10286402; doi:10.1186/s12906-023-04042-4)

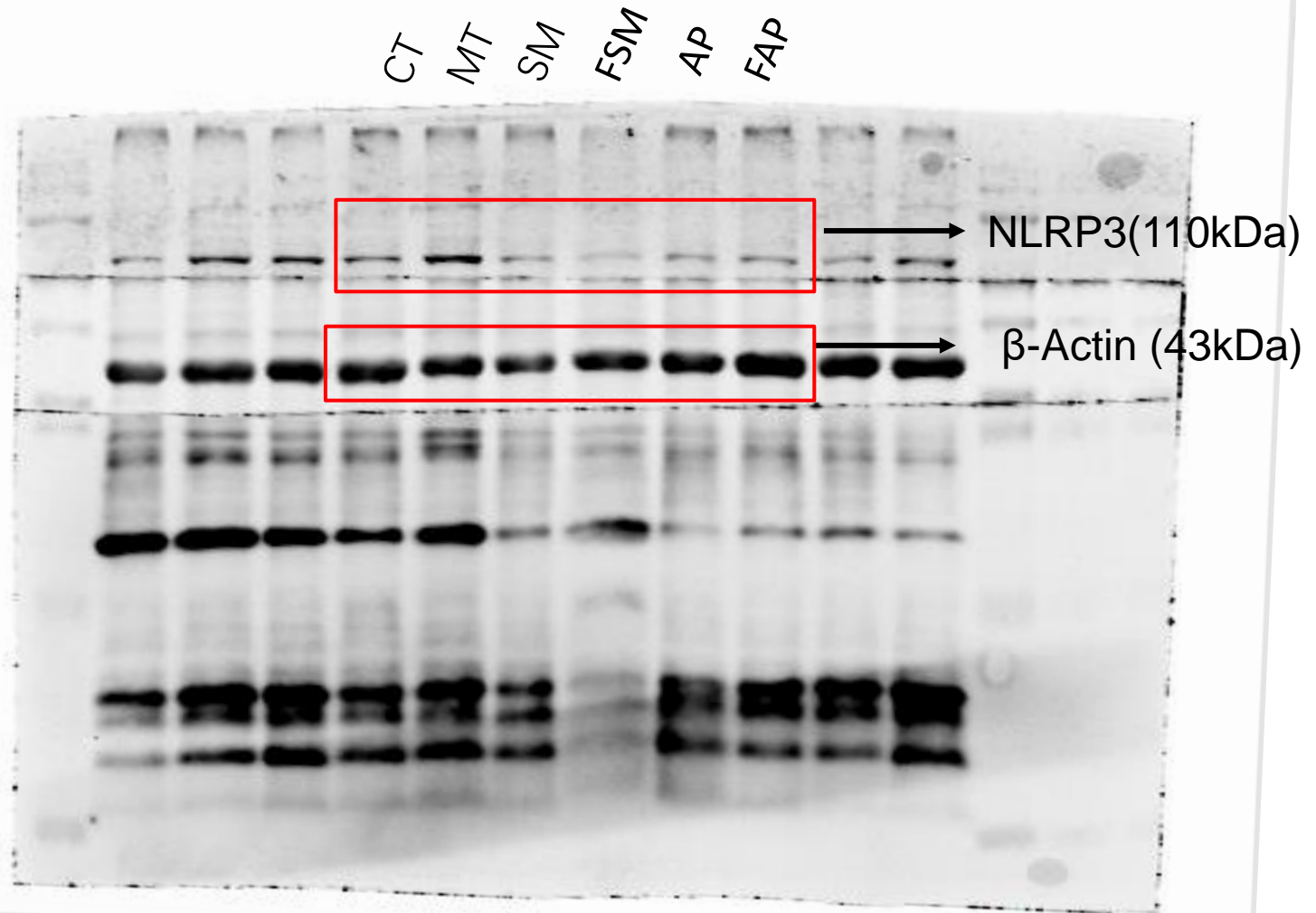

CT MT SM FSM AP FAP

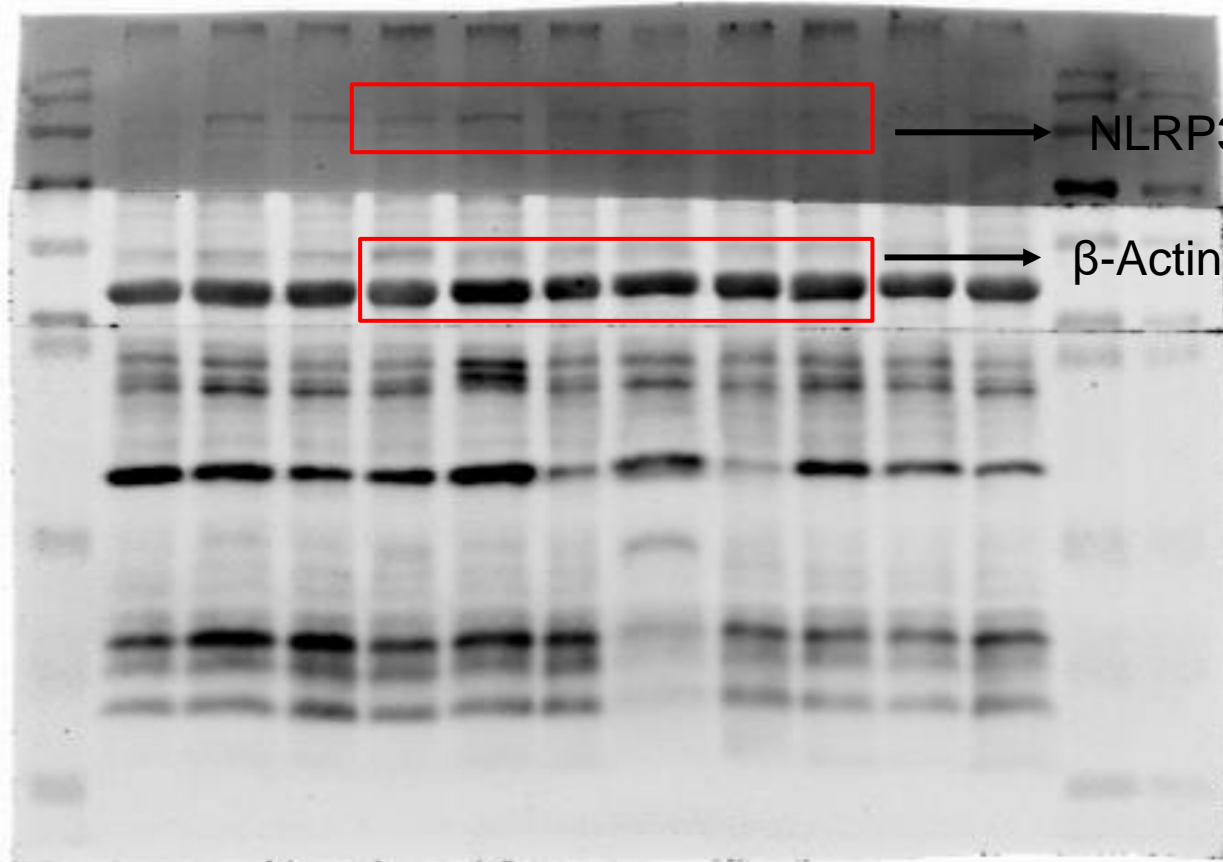

NLRP3(110kDa)

β-Actin (43kDa)

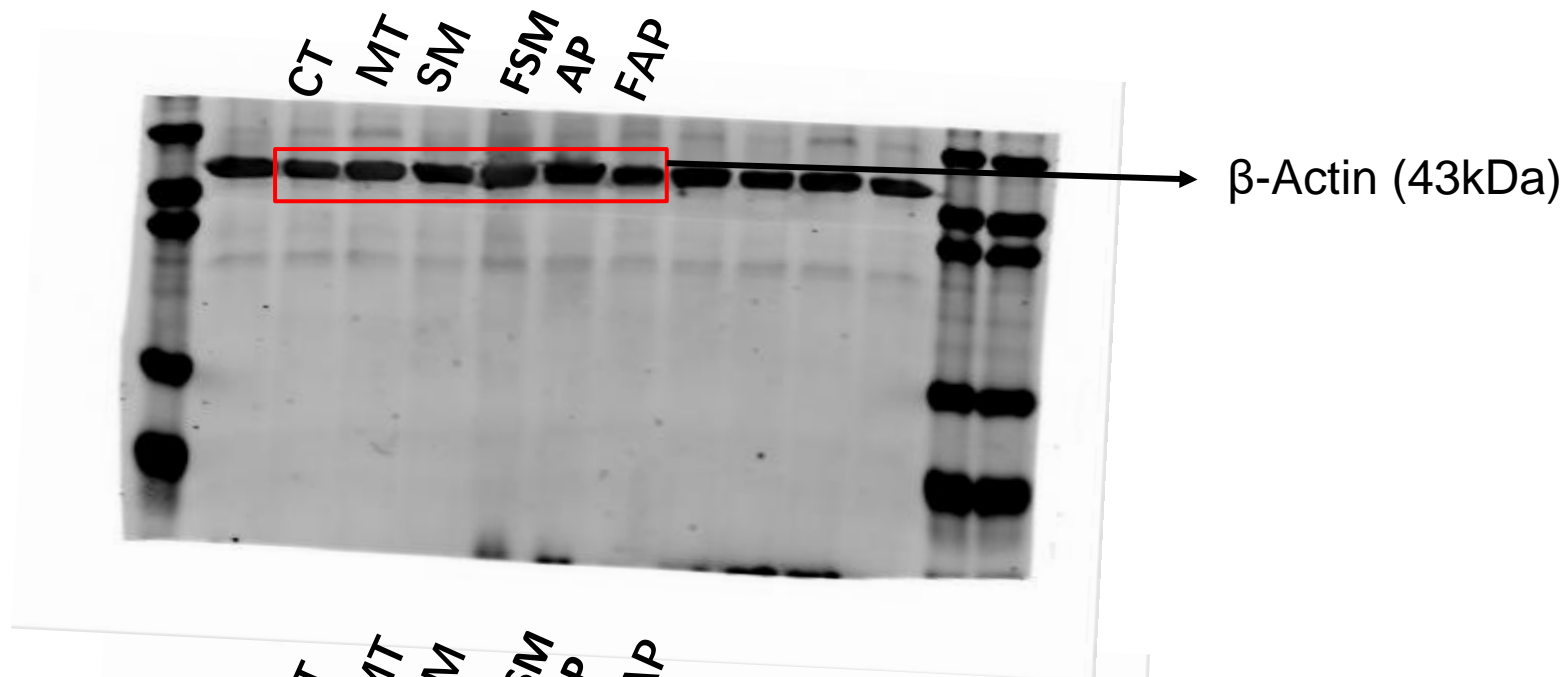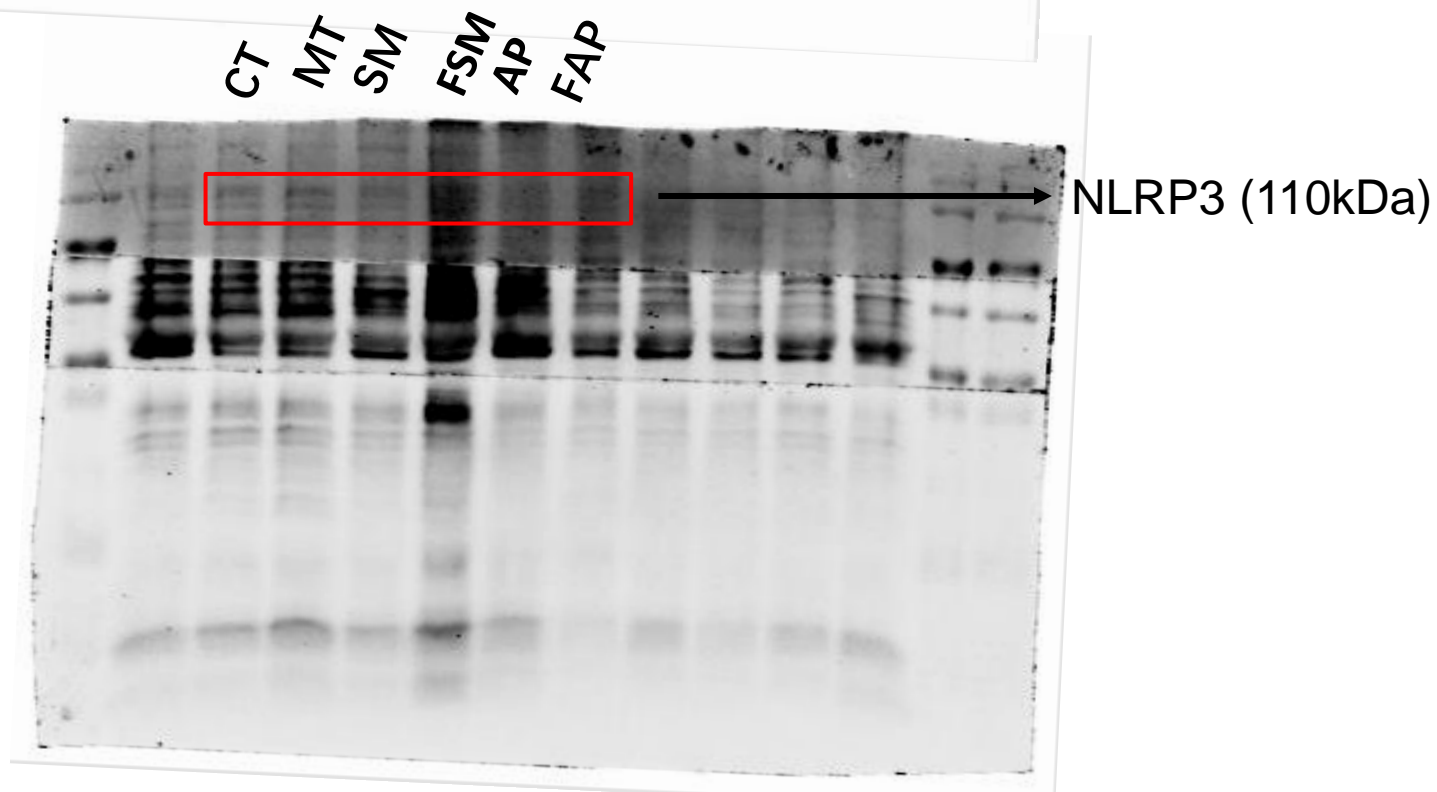

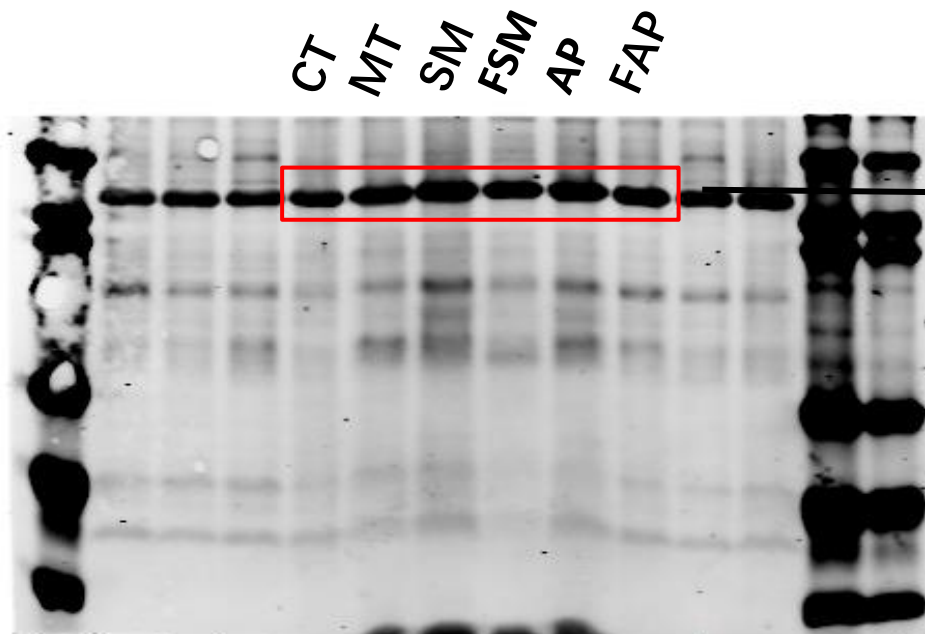

$\beta$ -Actin (43kDa)

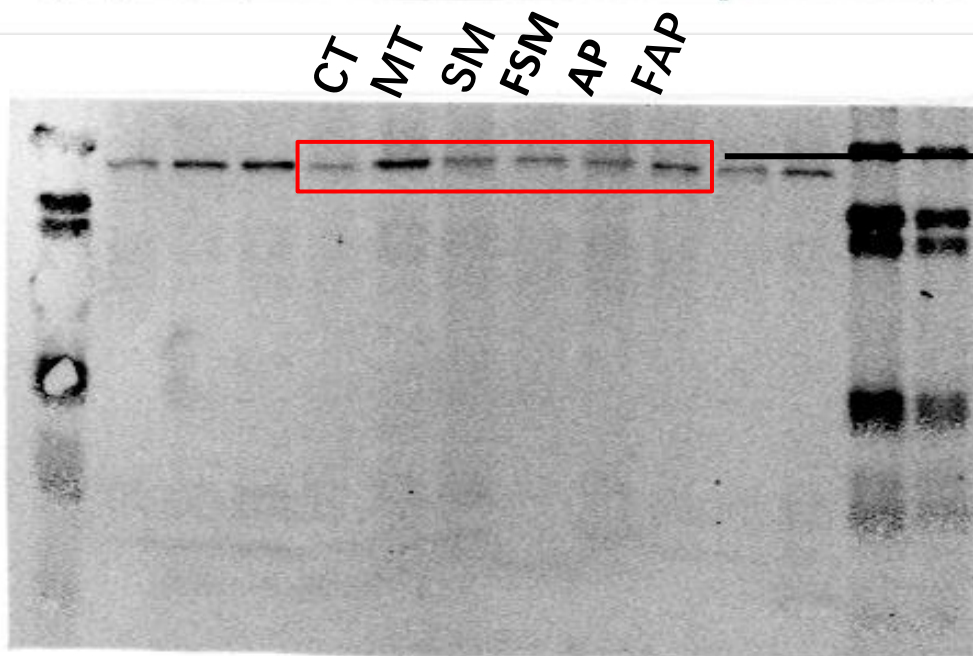

Caspase-1(45kDa)

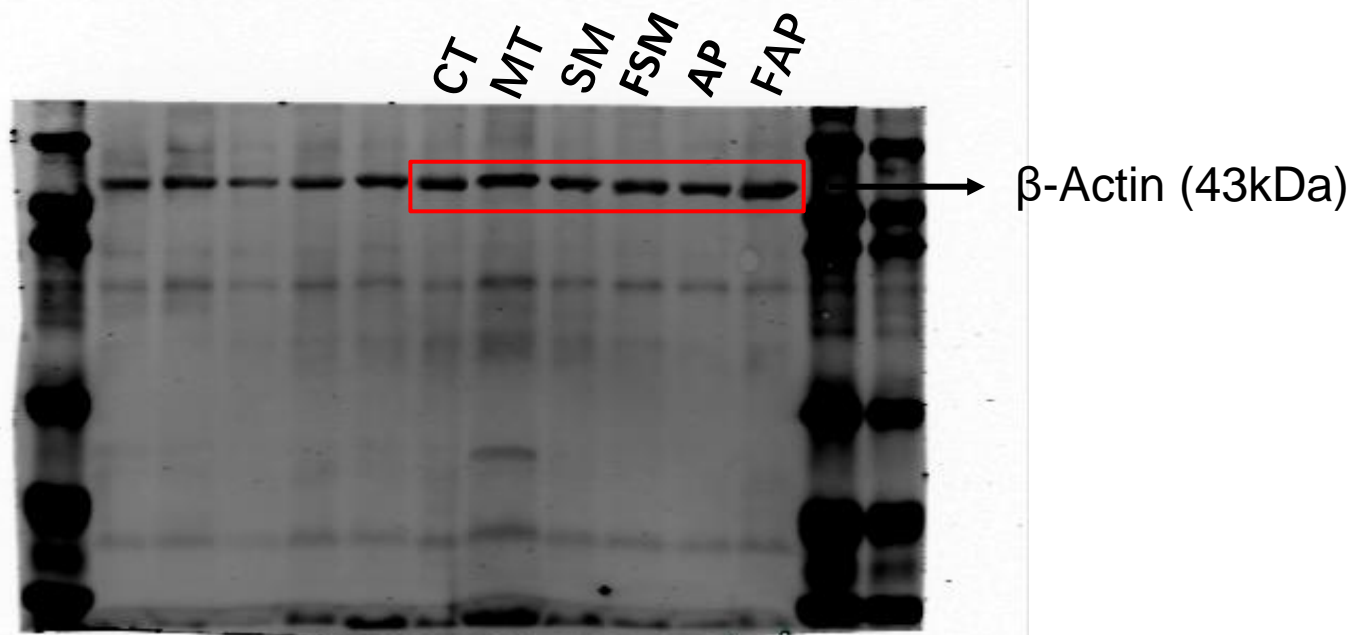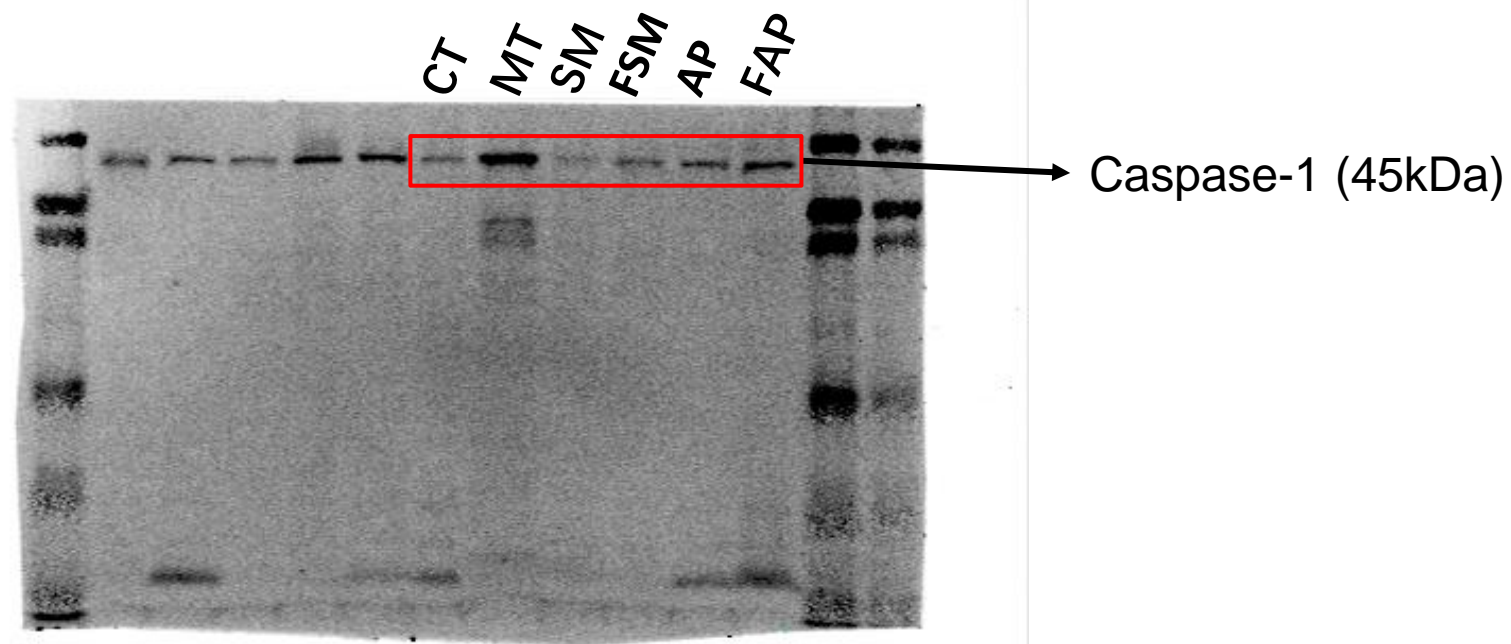

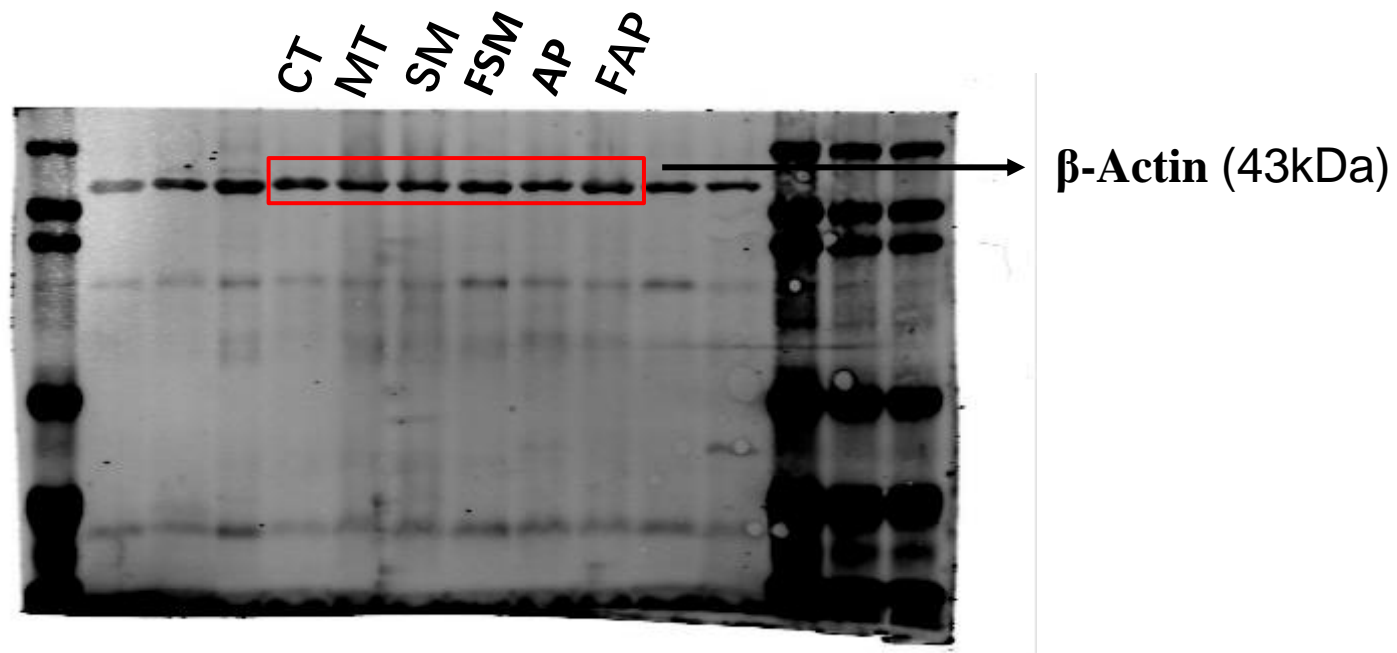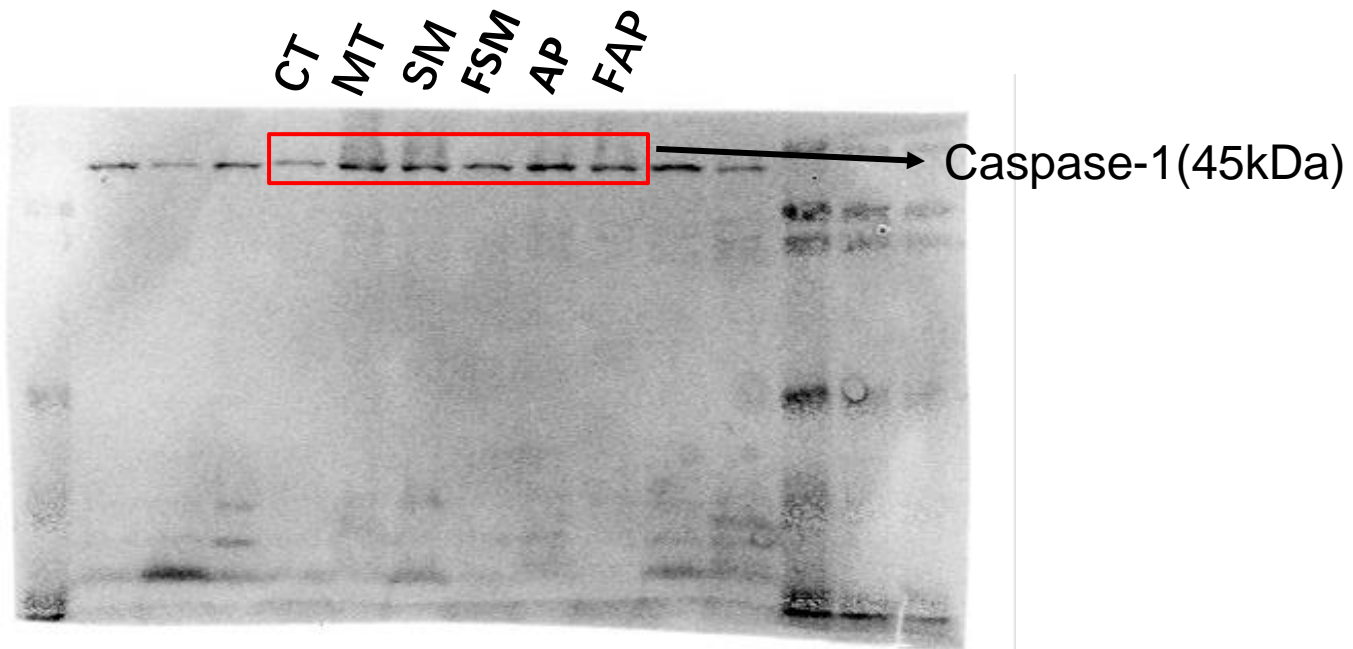

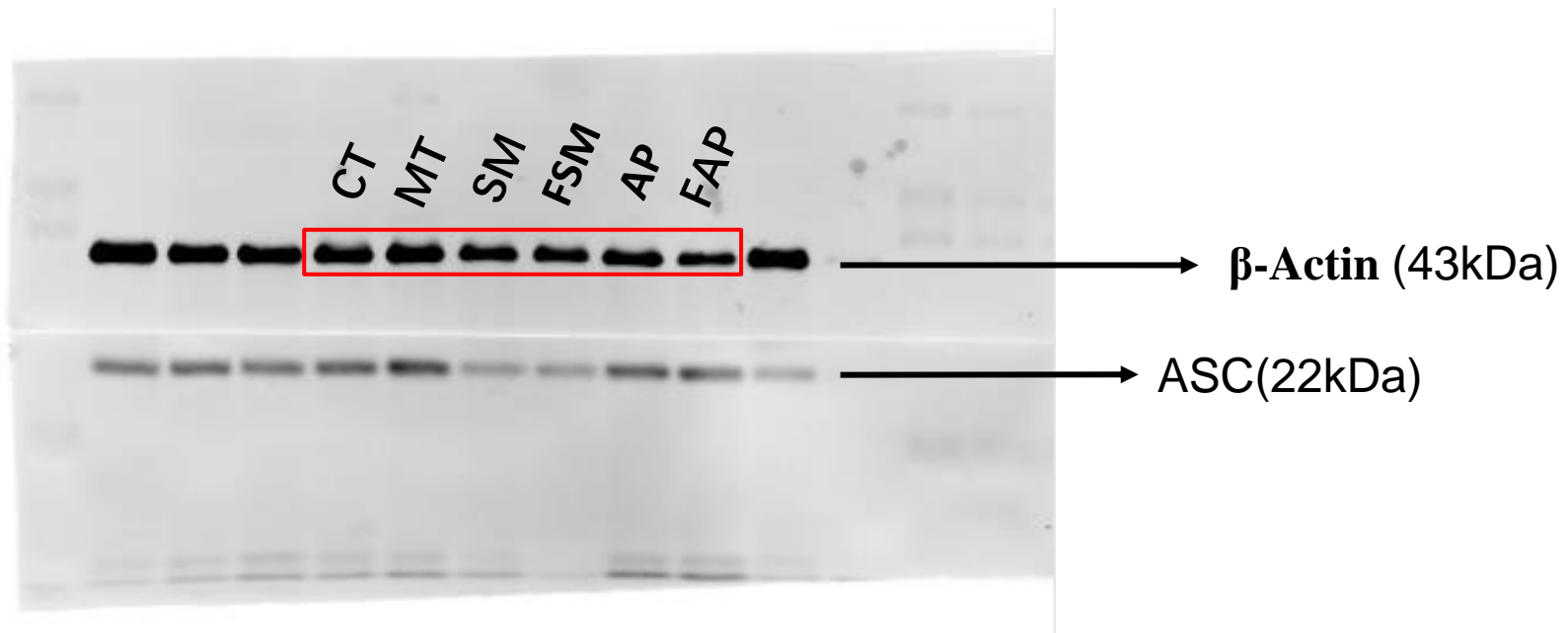

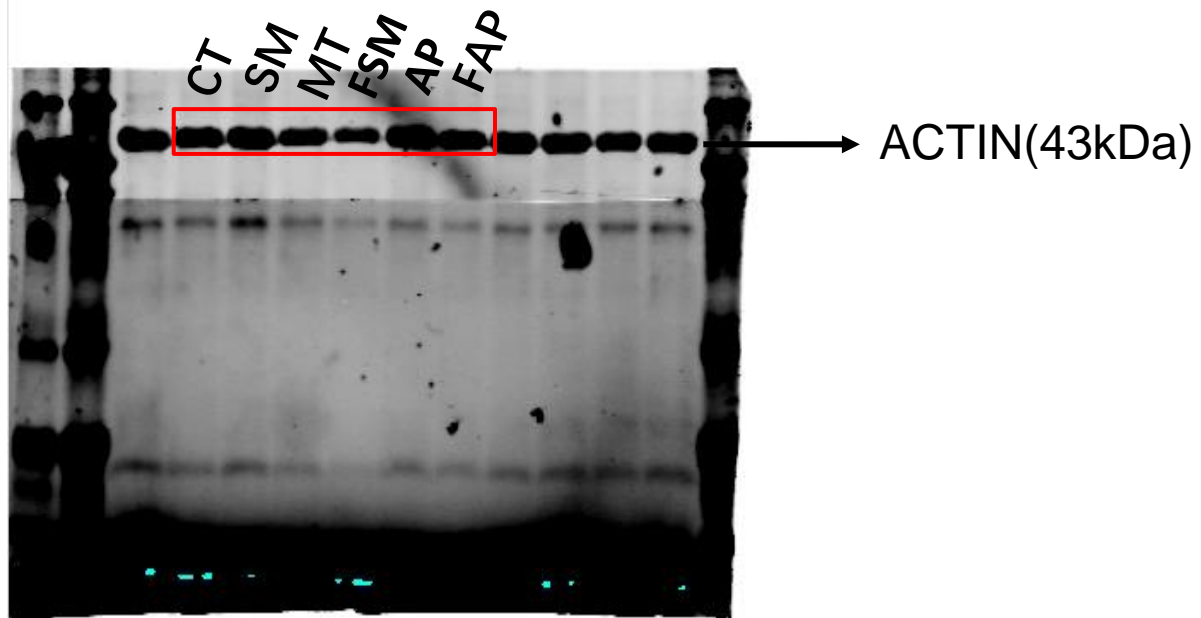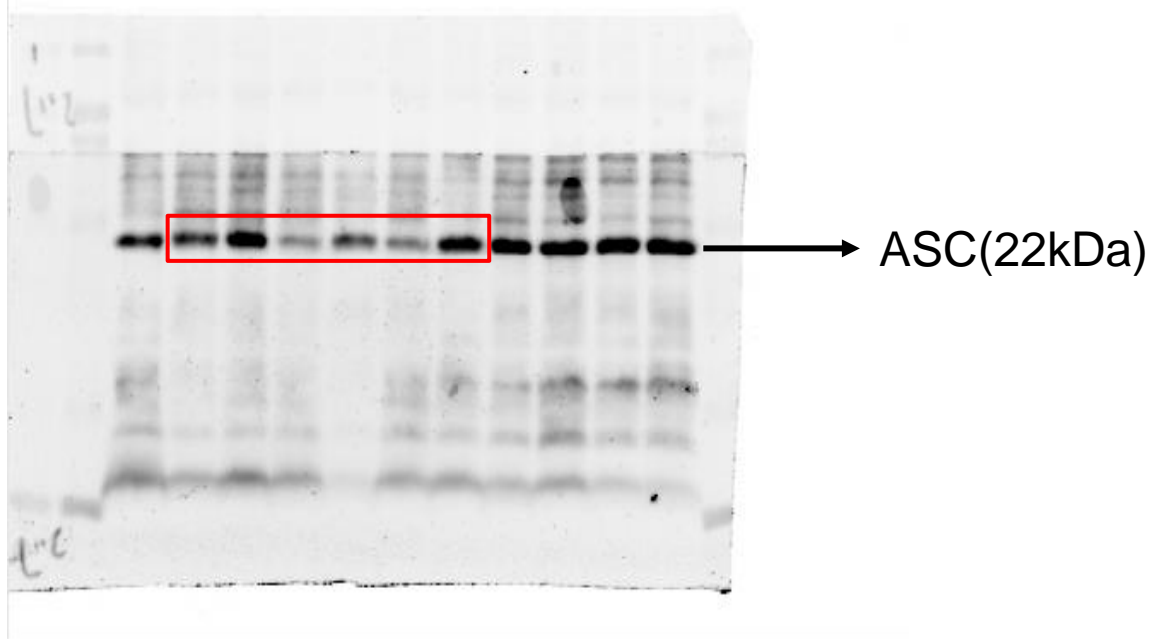

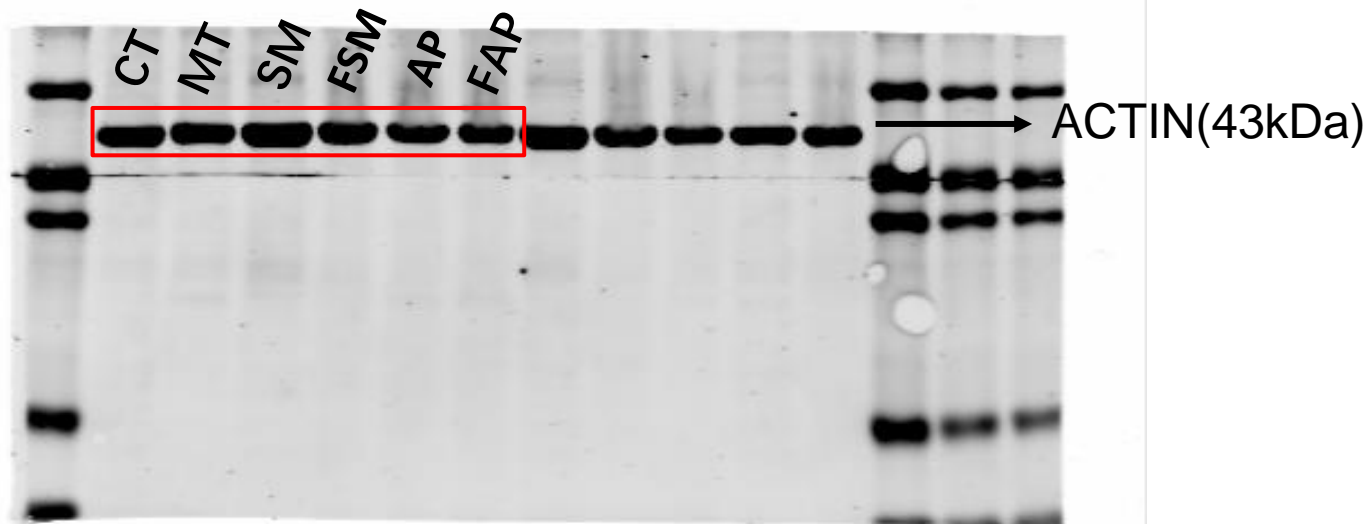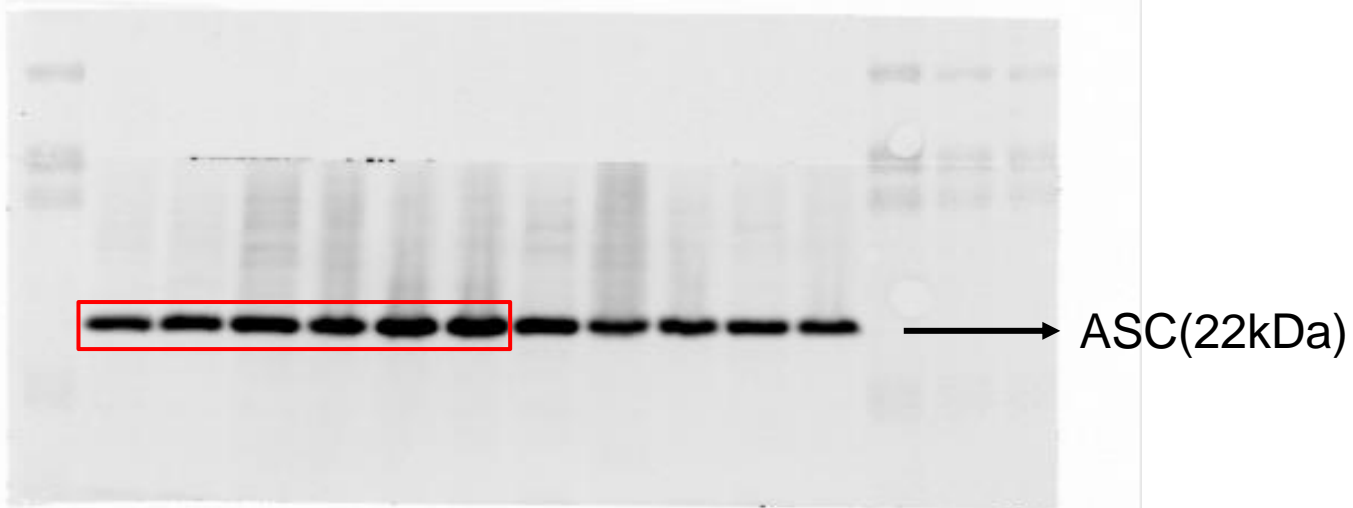

Supplement: Supplementary file 1 — Supplementary Material 1 [file 12906_2023_4042_MOESM1_ESM.pdf]
